# Luteolin ameliorates hyperuricemic nephropathy by activating urate excretion and Nrf2/HO‐1/NQO1 antioxidant pathways in mice

**DOI:** 10.1002/fsn3.4403

**Published:** 2024-08-20

**Authors:** Huifan Yu, Linsheng Huang, Lili Gui, Zhengkun Wu, Han Luo, Mao Xu, Yan Zhang, Yongshuai Qian, Wenjie Cao, Li Liu, Fei Li

**Affiliations:** ^1^ School of Pharmaceutical Sciences, Hubei Key Laboratory of Wudang Local Chinese Medicine Research Hubei University of Medicine Shiyan Hubei China; ^2^ Institute of Biomedicine Hubei University of Medicine Shiyan Hubei China; ^3^ Department of Hepatopancreatobiliary Surgery, Taihe Hospital Hubei University of Medicine Shiyan Hubei China

**Keywords:** hyperuricemic nephropathy, KIM‐1, luteolin, Nrf2, uric acid transporters

## Abstract

Luteolin is a natural flavonoid, which exists in many plants, including onions, broccoli, carrots, peppers, celery, olive oil, and mint. Luteolin is a dietary flavonoid with potent uric acid‐lowering and antioxidant bioactivities. To date, the mechanism by which luteolin alleviates hyperuricemia nephropathy (HN) still needs to be better defined. This study aims to evaluate the therapeutic efficacy of luteolin in a preclinical mouse model and in vitro. Luteolin was administered in the HN mice induced by the combination of potassium oxonate and hypoxanthine to evaluate the potential renoprotective effects in vivo. The NRK‐52E cells were stimulated with adenosine for in vitro evaluation. Hematoxylin and eosin staining, biochemical analysis, immunoblotting, immunofluorescence, and immunohistochemistry were performed for the histopathologic and mechanistic investigations. The results suggest that luteolin attenuated tubular dilation and epithelial atrophy in the renal tissue of HN mice. Further, luteolin improved biochemical indicators concerning renal functions and oxidative stress in vivo. Mechanistically, luteolin reduced the renal expressions of KIM‐1 and caspase‐3. Luteolin activated renal SIRT1/6 cascade and its downstream Nrf2‐mediated antioxidant pathway. Furthermore, luteolin elevated the renal expressions of ATP‐binding cassette subfamily G isoform 2 protein (ABCG2) and organic anion/cation transporters. In addition, livers of luteolin‐treated HN mice exhibited robust inhibition of xanthine oxidase. Together, our study shows that luteolin alleviates renal injury in the HN mice by activating urate excretion and Nrf2/HO‐1/NQO1 antioxidant pathways and inhibiting liver xanthine oxidase activity. Thus, luteolin may be a potential agent for the treatment of HN.

## Graphical abstract

This study provides mechanistic insights into how luteolin suppresses renal injury caused by hyperuricemic nephropathy (HN). Luteolin intervention significantly improved renal injury in HN mice. Through in vivo and in vitro experiments, we found that luteolin can significantly activate uric acid excretion and Nrf2/HO1/NQO1 antioxidant pathway and inhibit the activity of xanthine oxidase in the liver.

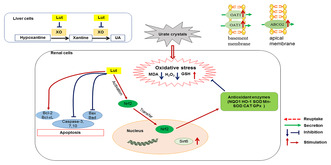

## 1 INTRODUCTION

Hyperuricemia (HUA) is a metabolic syndrome characterized by elevated blood uric acid levels due to abnormalities in purine metabolism or impaired excretion of uric acid (Yanai et al., 2021). The occurrence of hyperuricemia has seen an uptick over time, with a prevalence of 14.0% among Chinese adults, and the highest rates observed in males between the ages of 18 and 29 (C. Zhang et al., 2021). HUA is strongly associated with gout, chronic kidney disease, hypertension, insulin resistance, and cardiovascular disease, acting as a separate risk factor for the development of these conditions (Perticone et al., 2012). Under typical physiological conditions, approximately 70% of uric acid is eliminated via the renal excretory system (Levinson & Sorensen, 1980). However, when the uric acid load exceeds the clearance capacity of the kidneys, uric acid crystals tend to deposit in the collecting ducts, renal pelvis, and urethra, resulting in intrarenal and extrarenal obstruction, which eventually leads to hyperuricemic nephropathy (HN) (Johnson et al., 2018).

Under physiological conditions, uric acid is produced and excreted to maintain a dynamic balance. Hyperuricemia is easily triggered when uric acid production increases or uric acid excretion decreases. Uric acid is an insoluble end product of purine metabolism in humans. In the presence of nucleotidases, purine nucleotides are dephosphorylated to form purine nucleosides. Purine nucleosides and purines are further converted to uric acid by the rate‐limiting enzyme xanthine oxidase (XO) (Filippatos et al., 2011). Thus, abnormal purine metabolism is a possible molecular mechanism underlying HN.

Uric acid metabolism comprises four steps: filtration, reabsorption, secretion, and post‐secretion reabsorption. After its glomerular filtration into the renal tubules, uric acid transport proteins play an essential role in the subsequent metabolic processes. They are divided into two major groups: reabsorption and secretion proteins. The uric acid reabsorption proteins, uric acid anion transporter 1 (URAT1) and glucose transporter 9 (GLUT9), facilitate the reabsorption of uric acid into the blood (Ichida, 2009; Matsuo et al., 2008). The uric acid secretory protein organic anion transporter family (OATs) members, OAT1 and OAT3, and the ATP‐binding cassette superfamily G member 2 (ABCG2) promote the secretion of uric acid into the urine before excretion (Drabkin et al., 2019; Hoque et al., 2020). Abnormal secretion and reabsorption of uric acid in the kidneys underlie the pathogenesis of hyperuricemia. Uric acid transport proteins play crucial roles in the uric acid metabolic pathway and are compelling entry points for the treatment of HN.

In this study, uric acid was found to have a dual effect, scavenging superoxide, hydroxyl radicals, and singlet oxygen on the one hand, thus acting as an antioxidant (Horsfall et al., 2014). In contrast, at lower levels of other antioxidants or higher levels of uric acid, it acts as an oxidation promoter (So & Thorens, 2010). Hyperuricemia is often accompanied by significant oxidative stress damage (Cristobal‐Garcia et al., 2015). Nuclear factor erythroid 2‐related factor 2 (Nrf2) is an oxidative stress‐hypersensitive transcription factor that maintains cellular redox homeostasis. Under normal physiological conditions, the Kelch‐like ECH‐associated protein 1 (Keap1) anchors Nrf2 to the cytoplasm. When cells receive oxidative stress signals, Nrf2 rapidly dissociates from Keap1, translocates to the nucleus, and binds to antioxidant response elements (ARE) to promote the transcription of antioxidant enzyme genes, such as quinone oxidoreductase 1 (NQO1) (Pfefferle et al., 2020). The Sirtuin family is an essential regulator of various physiological activities, such as cellular metabolism, aging, apoptosis, inflammatory responses, and oxidative stress (Sosnowska et al., 2017). SIRT1 and SIRT6 are known to be involved in the dissociation of the nuclear Nrf2‐Keap1 complex (Pan et al., 2016; Sourbier et al., 2014). Thus, lowering uric acid levels in patients with hyperuricemia may alleviate oxidative stress damage in the body.

At present, HUA treatment begins with the inhibition of uric acid production or promotion of its clearance, with commonly used drugs including benzbromarone (Ben), allopurinol, and febuxostat (Feb). However, these have serious adverse effects and can cause liver damage and failure (Lee et al., 2008). Therefore, there is a clear need for novel uric acid‐lowering drugs with a high efficacy and fewer side effects.

Luteolin, a natural flavonoid present in many plants, including onions, broccoli, and mint, has a broad spectrum of pharmacological effects, including anti‐inflammatory, antitumor, and neuroprotective (Boeing et al., 2020). Multispectral techniques combined with molecular docking methods have shown that luteolin competitively binds and inhibits XO activity (Yan et al., 2013). In addition, luteolin has a powerful uric acid‐lowering function (Kim et al., 2021). However, the direct protective effects of luteolin against HN have not yet been demonstrated. In this study, an adenosine‐ and XO‐induced NRK‐52E cell model and a mouse model of HN were established via the intraperitoneal injection of potassium oxalate combined with hypoxanthine to evaluate the hypouricemic and nephroprotective effects of luteolin.

The results showed that luteolin ameliorates UA‐induced kidney injury and apoptosis, promotes uric acid excretion, and inhibits hepatic XO activity. This study is the first to propose that luteolin alleviates renal injury by activating the Nrf2‐mediated endogenous antioxidant system, indicating that the natural flavonoid lignocaine is beneficial for the treatment of hyperuricemia‐induced kidney damage.

## 2 MATERIALS AND METHODS

### 2.1 Reagents and instruments

Benzbromarone, febuxostat, and luteolin were obtained from Yichang Changjiang Pharmaceutica (Hubei, China), Huazhong Haiwei (Beijing, China), and Baoji Chenguang (Shaanxi, China), respectively. Oxoxazine potassium salt and hypoxanthine were obtained from Sigma‐Aldrich (St. Louis, MO). A protease inhibitor cocktail was obtained from AMRESCO (Solon, OH), and a BCA kit was obtained from Thermo (Waltham, MA). Applygen Technologies Inc. (Beijing, China) provided a pre‐stained protein molecular weight standard.

### 2.2 Animals and treatment

The techniques employed for animal treatment were delineated in our prior investigation (Yu et al., 2021). All the animal care and experimental procedures were conducted by the Chinese Association for Laboratory Animal Science's “Guide for the Care and Use of Laboratory Animals,” based on the principle of laboratory animal care (People's Republic of China National Standard GB/T 35892‐2018). The Animal Welfare Committee approved animal studies at the Hubei University of Medicine (SYXK2019‐0031). SPF‐grade ICR male mice originated from Beijing Weitong Lihua Laboratory Animal Technology Co., Ltd [certificate number: SCXK (Beijing) 2016‐0006]. All animals were raised in rooms equipped with 12 h of light and 12 h of darkness each, having freedom of food and water access.

70 ICR male mice were randomized into 7 groups as follows: solvent control, model, benzbromarone (20 mg/kg) and febuxostat (10 mg/kg), and luteolin low‐, medium‐, and high‐dose groups (10, 30, and 90 mg/kg). On the first to the seventh day, administration to mice was performed by gavage at 18:00 daily. The solvent control, mode, and positive drug group were administered saline, and the corresponding doses of luteolin were given to the low, medium, and high groups. On the eighth day, normal saline was injected intraperitoneally into the solvent control group at noon each day. The other groups were injected intraperitoneally with potassium oxalate and hypoxanthine solution (300 mg/kg). Meanwhile, the solvent control and model groups were treated daily at 18:00 by intragastric instillation of normal saline, and the other groups were given the corresponding treatment for 7 days. Intragastric administration and intraperitoneal injection of each group mentioned above were performed at a dose of 0.4 mL/20 g. Subsequent animal sacrifice was performed by the welfare norms and animal‐based experimental principles.

The blood samples were collected 2 h later after the last instillation. The samples were centrifuged at a speed of 4000 rpm for a duration of 10 min at a temperature of 4°C. The resulting liquid was collected and preserved at a temperature of −80°C until it was examined.

### 2.3 Tissue extraction and fixation

Remove two kidneys from each mouse and scale them to calculate the renal index (organ index % = organ weight/final weight × 100), strip the outer membrane of either kidney, cut the middle part of the kidney into pieces, and fix them in 4% paraformaldehyde for histopathological observation. Other kidney tissues were stored in a −80°C refrigerator.

### 2.4 Determination of biochemical parameters

The lyophilized kidney and liver tissue samples were homogenized and centrifuged to obtain supernatants for subsequent experimental manipulation. The lyophilized kidney and liver tissue samples were homogenized and centrifuged to obtain supernatants for subsequent experimental manipulation.

The measurement of uric acid (UA), renal glutathione (GSH), and hydrogen peroxide (H_2_O_2_) was conducted using commercially available kits obtained from NJJC Biotechnology (Jiangsu, China), following the instructions provided by the manufacturer. Kits for assessing levels of renal glutathione peroxidase (GPx) and Mn‐superoxide dismutase (Mn‐SOD) were provided by Beyotime Biotechnology, located in Shanghai, China. Microalbuminuria (MUA) was assessed using an ELISA kit obtained from Wuhan New Qidi Biotechnology Co. Ltd (Hubei, China). According to the operating procedures supplied by the manufacturer, XO activity in serum and liver tissues was assessed using the commercially available kit obtained from NJJC Biotechnology (Jiangsu, China).

### 2.5 TUNEL staining

To perform TUNEL staining, utilize the in situ apoptosis detection kit obtained from Beyotime Biotechnology (Shanghai, China) and adhere to the guidelines provided by the manufacturer. Observe the staining results with a laser confocal fluorescence microscope (Olympus, FV3000RS, Japan).

### 2.6 Immunohistochemistry (IHC) and immunofluorescence staining

The detailed IHC staining techniques were elucidated in our previous scholarly investigation (Yu et al., 2021). In brief, immobilized kidney tissues were sliced into 4‐μm‐thick wax sections and hydrated after dewaxing. Using antibodies against KIM‐1 (1:300) for immunoperoxidase staining and visualizing with a bright‐field microscope (Olympus, BX53).

Fresh murine kidney tissues were hydrated in a 30% sucrose solution and immobilized in 4% paraformaldehyde for immunofluorescence studies. The fixed tissue was embedded with tissue OCT‐freeze medium and sliced with a microtome (4 μm). Briefly, the slices were put in a dark box that was humidified and kept at a temperature of 37°C for 1.5 h. During this time, the corresponding primary antibody (diluted 1:50) was added, followed by a 1‐h incubation with either goat anti‐rabbit IgG (Alexa Fluor® 488‐conjugated) or goat anti‐mouse IgG H&L (Alexa Fluor® 594‐conjugated) secondary antibodies (both diluted 1:50). The kidney tissue's nuclei were stained using 4,6 diamidino‐2‐phenylindole (DAPI, Beyotime, C1005) for a duration of 10 min. Subsequently, the tissue slices were examined under laser confocal fluorescence microscopy (Olympus, FV3000RS) at a magnification of 1000×. Table S1 displays all the antibodies utilized.

### 2.7 Western blot analysis

Freshly dissected kidney tissue was homogenized using a RIPA lysis buffer (Cell Signaling, #9806) and commercially available kits (SC003, Invent Biotechnologies, Inc., Beijing, China) to extract total, cytoplasmic, and nuclear protein, respectively. 20–40 μg of protein was denatured in the lysate at 95°C, followed by isolation using SDS‐PAGE gel electrophoresis, and subsequently transferred onto the PVDF membrane. The membrane was covered with 5% skimmed dry milk for 1 h and then left overnight at 4°C with the primary antibody. After 24 h, the membrane was exposed to the suitable HRP‐linked secondary antibody for a duration of 1.5 h. After rinsing, the membrane was exposed to a chemiluminescent detection reagent (Applygen, P1050‐250). The chemiluminescence signal was captured using a gel imaging system (GE, ImageQuant LAS 4000). All antibodies used are shown in Table S1.

### 2.8 Molecular docking

Simulating the binding modes of luteolin with Cattle XO (PDB entry: 2E1Q) by molecular docking, luteolin was inserted into binding sites in the crystal structures. In the catalyst software, we used a 2D/3D editor sketcher to design the luteolin's layout, minimizing its energy consumption. We selected the best complex based on default docking parameters based on the total docking score.

### 2.9 Microscale thermophoresis assay

To measure the binding affinity of luteolin with XO in vitro using a NanoTemper/Monolith NT.115 instrument (NanoTemper, Germany) based on MicroScaleThermophoresis (MST) technology. Briefly, the purified XO protein (molecular mass 160 kDa, M001) was lipidated with the RED‐NHS (NanoTemper Technologies), a protein labeling kit purchased from NJJC Biologicals. Label the protein in a dark environment at a temperature of 25°C for 30 min using a labeling buffer with a protein concentration of 10 μmol/L, maintaining a ratio of 3 parts dye to 1 part protein. Using a detergency cartridge balanced with MST buffer (50 mmol/L Tris–HCl pH 7.8, 150 mmol/L NaCl, 10 mmol/L MgCl_2_), the paint that had not reacted was eliminated. Typically, the labeling kit achieves a labeling degree of 0.8 as measured by UV/Visible spectrophotometry at 650 and 280 nmol/L.

### 2.10 Cell culture

The NRK‐52E cells were acquired from the Cell Resource Center at the Shanghai Institutes for Biological Sciences, which is part of the Chinese Academy of Sciences. Routine cell culture was performed using a constant temperature incubator set at 37°C with 5% CO_2_. The DMEM medium was supplemented with 10% (*v*/*v*) fetal bovine serum, 1% glutamine, 1% sodium pyruvate, and 100 U/mL each of penicillin and streptomycin. The cell density reached about 80% for subsequent experimental processing.

### 2.11 Cytotoxicity analysis

96‐well plates were used to seed cells at a density of 5000 cells per well. After culturing in serum‐free medium for an additional 24 h, when the cell density reached 80% in each well, luteolin was dissolved in dimethyl sulfoxide (DMSO) with a concentration range of 0, 0.1, 1, 10, 50, 100, 200, 400, and 600 μmol/L. Various levels of luteolin were individually introduced into 96‐well plates and incubated for 24 h. Subsequently, Cell Counting Kit‐8 (CCK‐8) reagent was supplemented, and absorbance at 450 nm was assessed using a microplate reader (Molecular Devices, Spectra Max 190) to evaluate cell viability.

### 2.12 Adenosine‐induced hyperuric acid cell model

Inoculate NRK52E cells into each well of a 24‐well plate at a density of 1 × 10^5^ cells per well. Add 1 mL of DMEM (Dulbecco's modified eagle medium) containing 10% FBS (fetal bovine serum) and culture the cells in a constant temperature incubator at 37°C and 5% CO_2_ for 24 h. Once the cells reach 80% growth, switch to a serum‐free medium and continue the culture for another 24 h. Meanwhile, pre‐incubate the luteolin groups with 2, 10, and 50 μmol/L luteolin for 24 h, respectively. Afterward, each well was washed three times using PBS. Then, the model groups received the addition of 2.5 mmol/L adenosine in serum‐free medium, while the luteolin groups were incubated with serum‐free medium containing 2.5 mmol/L adenosine and luteolin at concentrations of 2, 10, and 50 μmol/L for 24 h. Following the 24‐h incubation period, each well was supplemented with 0.005 U/mL XO. After 6 h of treatment, the culture supernatant was gathered and the quantity of uric acid was assessed using HPLC.

### 2.13 HPLC analysis

The detection of uric acid in the cell supernatant was carried out using an HPLC system (Thermo Ultimate3000, USA) equipped with a diode array detector (DAD) and an auto‐injector for high‐performance liquid chromatography (HPLC). Agilent's Zorbax SB C18 columns (4.6 × 250 mm, 5 μm) were utilized to separate all desired analytes. The uric acid was dissolved with 0.02 mol/L sodium hydroxide and diluted to gradient concentrations (0.01, 0.02, 0.04, 0.08, 0.16, 0.32, 0.64 mg/mL). Mobile phase A consisted of 0.52 mmol/L sodium 1‐pentenesulfonate and 0.20 mol/L monopotassium phosphate. Adjust the pH of mobile phase A to 4.0 using phosphoric acid (HPLC grade) to ensure stability. Mobile phase B was acetonitrile (*v*/*v*). The mobile phase A: B ratio was 95:5. The rate of flow was 1.0 mL/min, while the wavelength of detection was 254 nm. Equilibrating the system for at least 30 min before injecting the first sample was necessary. The injection volume was 20 μL, and the duration of the run was 20 min. Keep the column temperature at 35°C.

### 2.14 Uric acid–induced hyperuric acid cell model

The NRK‐52E cells were incubated in 6‐well dishes for a duration of 24 hours. Following this, all the groups were switched to a medium without serum and incubated for 12 h. Afterward, each well was rinsed three times using PBS. The experimental groups underwent modifications, with the control + luteolin groups being exposed to 50 μmol/L luteolin for 24 h. Meanwhile, the model groups, luteolin groups, and luteolin + ML385 groups were exposed to 15 mg/mL uric acid for 24 h, 15 mg/mL uric acid combined with 50 μmol/L luteolin, and 15 mg/mL uric acid combined with 50 μmol/L luteolin + 10 μmol/L ML385, respectively. The kits were used to measure the cell viability, as well as the levels of MDA and GSH in the cells. Western blot analysis was performed to detect the protein expression levels of Nrf2, HO‐1, and NQO1.

### 2.15 ROS generation assay

The levels of intracellular ROS were measured using kits as per the instructions provided by the manufacturer (Solarbio, CA1410). NRK‐52E cells were seeded into 8 chambers at a concentration of 4 × 10^4^ cells per well and incubated at 37°C for 24 h. Following cell attachment, the cell grouping and processing procedures were identical to those used for MDA detection. Next, the cells were treated with 10 μmol/L DCFH‐DA and incubated for 20 min at room temperature in the absence of light. After incubation, the cells were washed with serum‐free medium three times. Fluorescence images were observed using a confocal microscope (Olympus FV3000RS).

### 2.16 Statistical analyses

We duplicated an experiment at least thrice. Mean ± standard deviation represents the data. The t‐test analyzed only two groups involved, while one‐way ANOVA analyzed multiple comparisons between groups. The *p* value <.05 was considered to be statistically significant for all the conducted tests.

## 3 RESULTS

### 3.1 Luteolin reduces uric acid levels and attenuates renal histopathological damage in mice with hyperuricemic nephropathy

**FIGURE 1:**
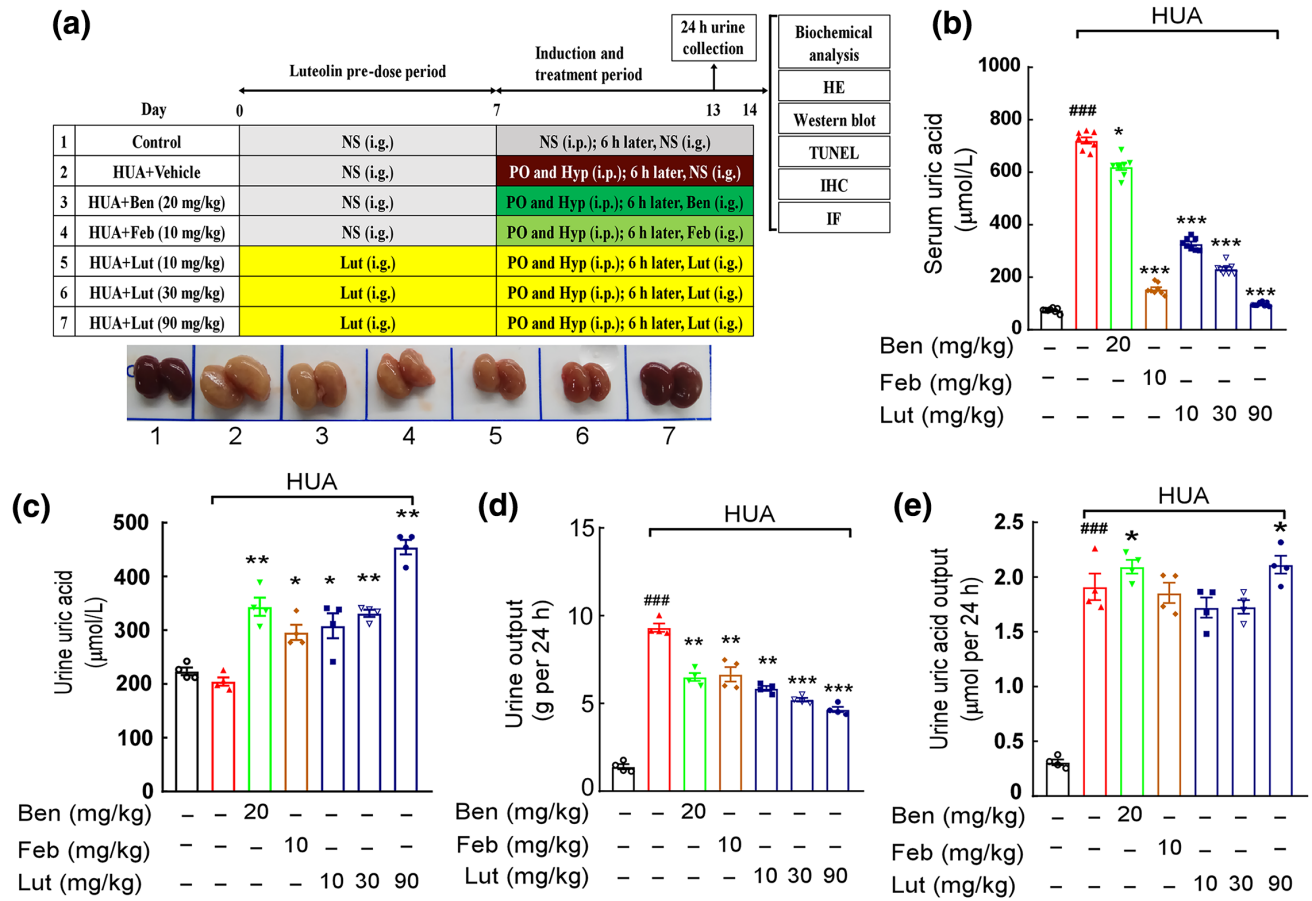
Luteolin reduces uric acid levels and attenuates renal histopathological damage in mice with hyperuricemic nephropathy. (a) Brief experimental scheme and representative kidney appearance of mice, from left to right (1–7), namely solvent control group, model group, benzbromarone group (20 mg/kg), febuxostat group (10 mg/kg), luteolin low‐, medium‐, and high‐dose groups (10, 30, 90 mg/kg). (b) Serum UA. (c) Urine UA. (d) 24 h urine output. (e) 24 h urine UA output. Data were means ± *SD* (*n* = 8 for a, *n* = 4 for c–e). ^###^ *p* < .001 *vs*. control group. *p < .05, **p < .01, ***p < .001 *vs*. HUA model group.

A model of hyperuricemic nephropathy was created using solutions of potassium oxalate and hypoxanthine, which exhibited the typical traits of increased levels of uric acid in the blood and impaired kidney function. On morphological grounds, the kidneys of HUA mice were significantly swollen, which improved after luteolin administration (Figure 1a). The results of our prior histological examination using hematoxylin and eosin staining revealed the presence of tubular dilatation and tubular epithelial atrophy in both the model and benzbromarone groups. Simultaneously, a discernible absence of comparison emerged between the luteolin medium and high‐dose cohorts and the solvent control group, thereby suggesting that luteolin within a specific dosage range possesses the potential to ameliorate the renal impairment induced by hyperuricemia (Yu et al., 2021). Throughout the study, the serum uric acid was also observed, and there was an elevation in the uric acid level in HUA mice (Figure 1b). Low, medium, and high doses of luteolin significantly reduced the serum uric acid concentration in HUA mice and showed dose‐dependent tolerance (Figure 1b). Meanwhile, medium and high doses of luteolin increased urinary uric acid concentrations compared to HUA mice (Figure 1c). The assay revealed a notable rise in the amount of urine excreted in a 24‐h period in HUA mice compared to the control group (Figure 1d). A high dose of luteolin reduced urine volume in HUA mice and could increase uric acid excretion in urine for 24 h (Figure 1e).

### 3.2 Luteolin administration improves renal dysfunction in mice with hyperuricemic nephropathy

**FIGURE 2:**
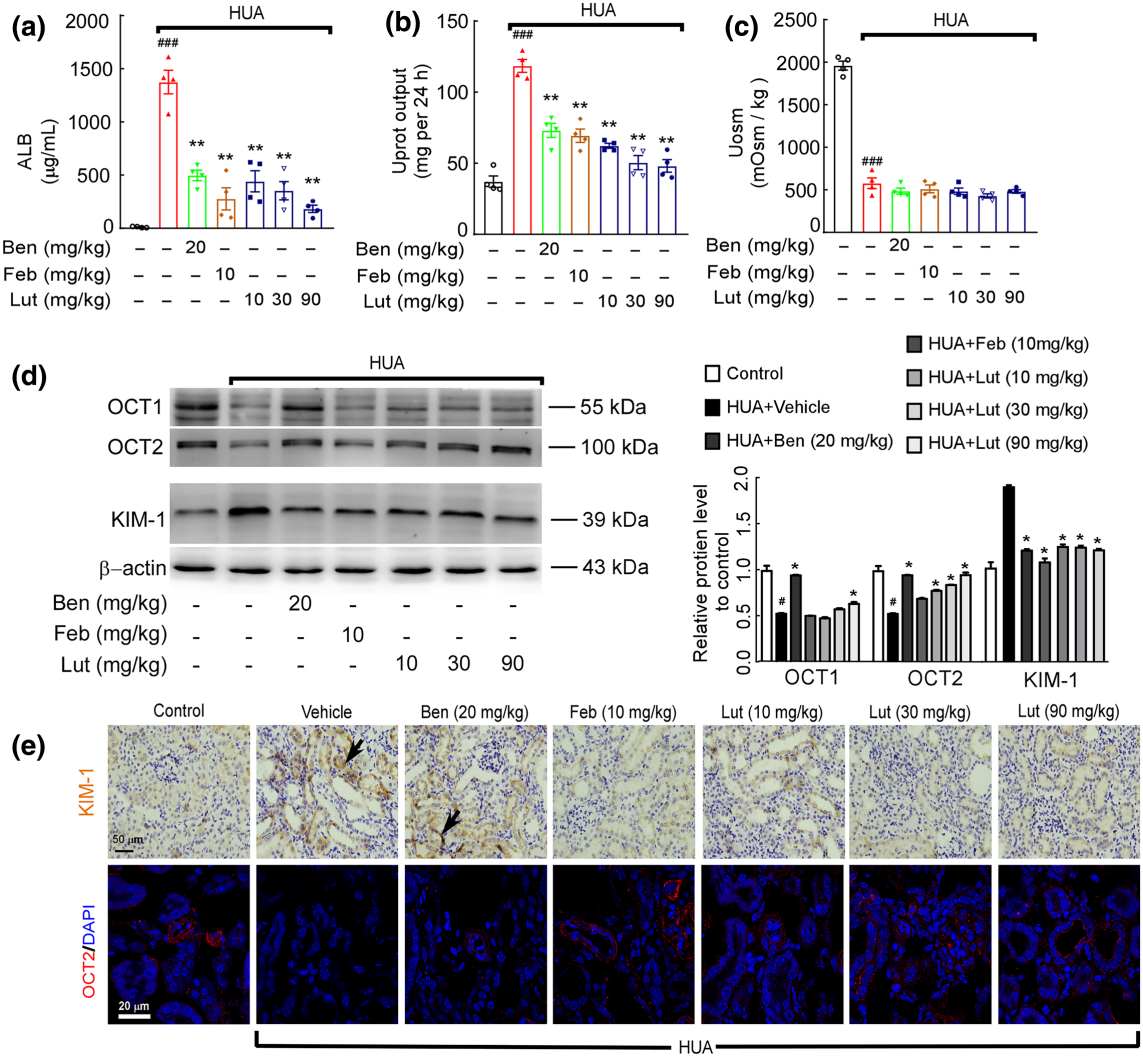
Luteolin administration improves renal dysfunction in mice with hyperuricemic nephropathy. (a) Urine microalbumin (ALB). (b) 24 h urine protein output. (c) Urine osmotic pressure. Data were means ± *SD* (*n* = 4 for a‐c, *n*). ^###^ *p* < .001 *vs*. control group. **p < .01 *vs*. HUA model group. (d) Protein expression level of OCT1, OCT2, and KIM‐1 in the kidney was determined by Western blots (left) and quantifications (right), which were normalized with *β*‐actin. Data were means ± *SD* (*n* = 3). ^#^ *p* < .05 *vs*. control group. *p < .05 *vs*. HUA model group. (e) Immunohistochemical detection of KIM‐1 expression in kidney tissue. Photomicrographs of OCT2 immunofluorescence stained kidney tissues in control and hyperuricemia mice with or without luteolin treatment (magnification 1000×).

According to our previous research results (Yu et al., 2021), the levels of serum BUN, serum creatinine, and urine microalbumin (Figure 2a) were increased in mice with nephropathy compared to controls, which was improved after luteolin intervention. Moreover, compared with the model group, luteolin significantly reduced 24 h urinary protein excretion in HUA mice on a dose‐dependent basis (Figure 2b). However, luteolin did not affect the decrease in urine osmolality caused by hypercalcemic nephropathy (Figure 2c). Furthermore, the HUA group exhibited an elevation in KIM‐1 protein levels in the kidney, while the levels of OCT1 and OCT2 proteins in the kidney were reduced compared to the solvent control group. However, this effect was reversed following the administration of lignocaine, as depicted in Figure 2d. Immunohistochemistry or immunofluorescence stained confirmed the above Western blot results (Figure 2e). These results demonstrated that luteolin treatment effectively ameliorated potassium oxalate and hypoxanthine‐induced renal insufficiency and pathological kidney injury in HUA mice.

### 3.3 Luteolin inhibits renal cell apoptosis in hyperuricemic mice

**FIGURE 3:**
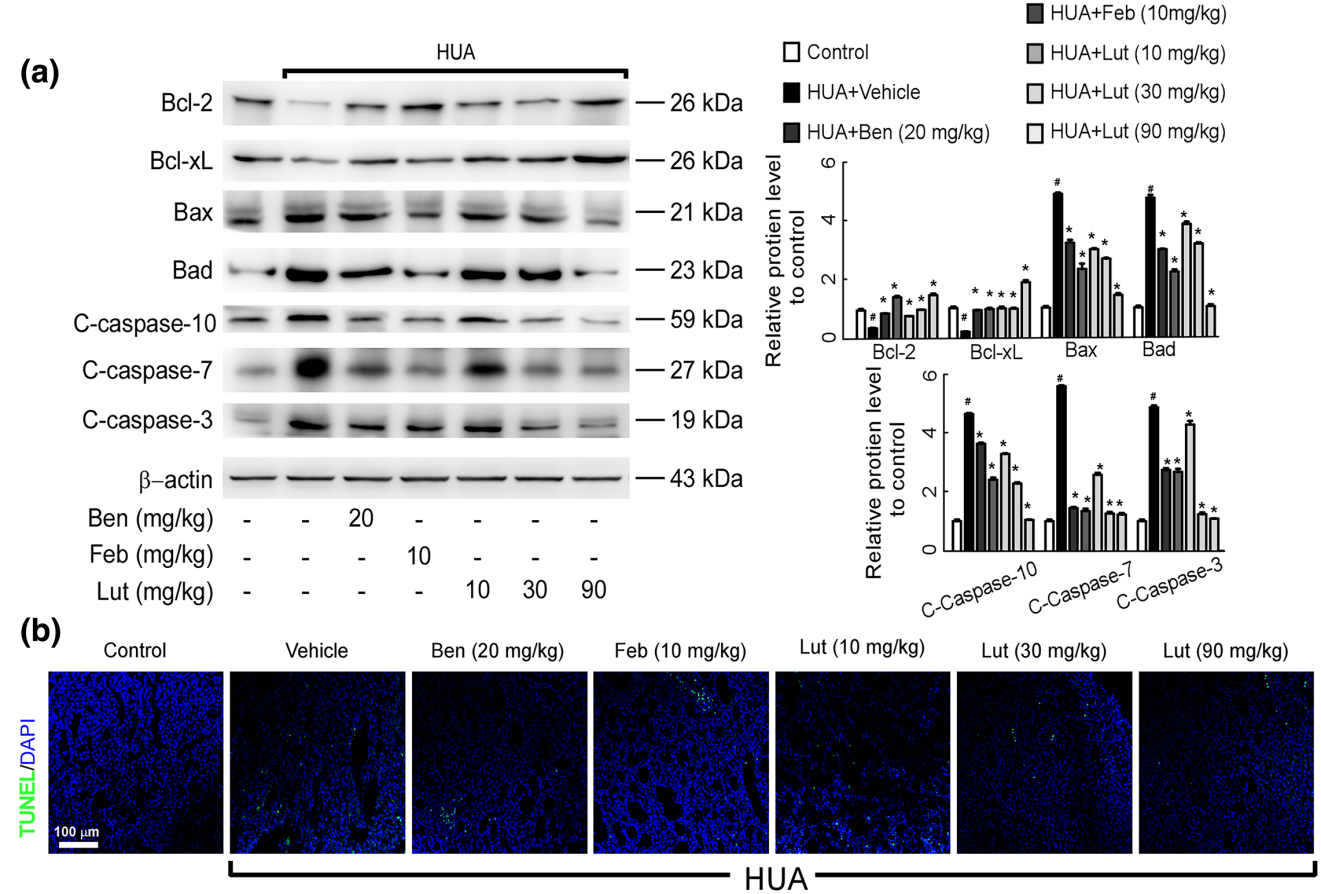
Luteolin inhibits renal cell apoptosis in hyperuricemic mice. (a) Protein expression levels of apoptosis‐related protein in the kidney were determined by Western blots (left) and quantifications (right), which were normalized with *β*‐actin. Data were means ± *SD* (*n* = 3). ^#^ *p* < .05 *vs*. control group. *p < .05 *vs*. HUA model group. Cleaved‐caspase is the activated form of caspase. C‐caspase‐3: Cleaved‐caspase‐3, C‐caspase‐7: Cleaved‐caspase‐7, C‐caspase‐10: Cleaved‐caspase‐10. (b) Photomicrographs of TUNEL immunofluorescence stained kidney tissues in control and hyperuricemia mice with or without luteolin treatment (magnification 200×).

Studies have found that high uric acid promotes apoptosis in kidney cells via multiple pathways. Here, we investigated the effects of luteolin on apoptosis‐related proteins in the kidneys of mice with hyperuricemic nephropathy. The Western blotting assay revealed that the model groups exhibited decreased levels of the anti‐apoptotic proteins Bcl‐2 and Bcl‐xL, in contrast to the solvent control. Additionally, there was an upregulation in the expression of pro‐apoptotic proteins including Bax, Bad, cleaved caspase‐10, cleaved caspase‐7, and cleaved caspase‐3. Luteolin effectively reversed the above phenomena (Figure 3a). TUNEL staining further verified that low, medium, and high doses of luteolin significantly inhibited kidney cell apoptosis (Figure 3b). Hence, our findings indicate that luteolin could potentially reduce uric acid levels in HUA mice by preventing cell death in kidney tissues. This is achieved by increasing the expression of proteins that anti‐apoptotic and decreasing the expression of proteins that pro‐apoptotic.

### 3.4 Luteolin alleviates renal oxidative stress in mice with hyperuricemic nephropathy

**FIGURE 4:**
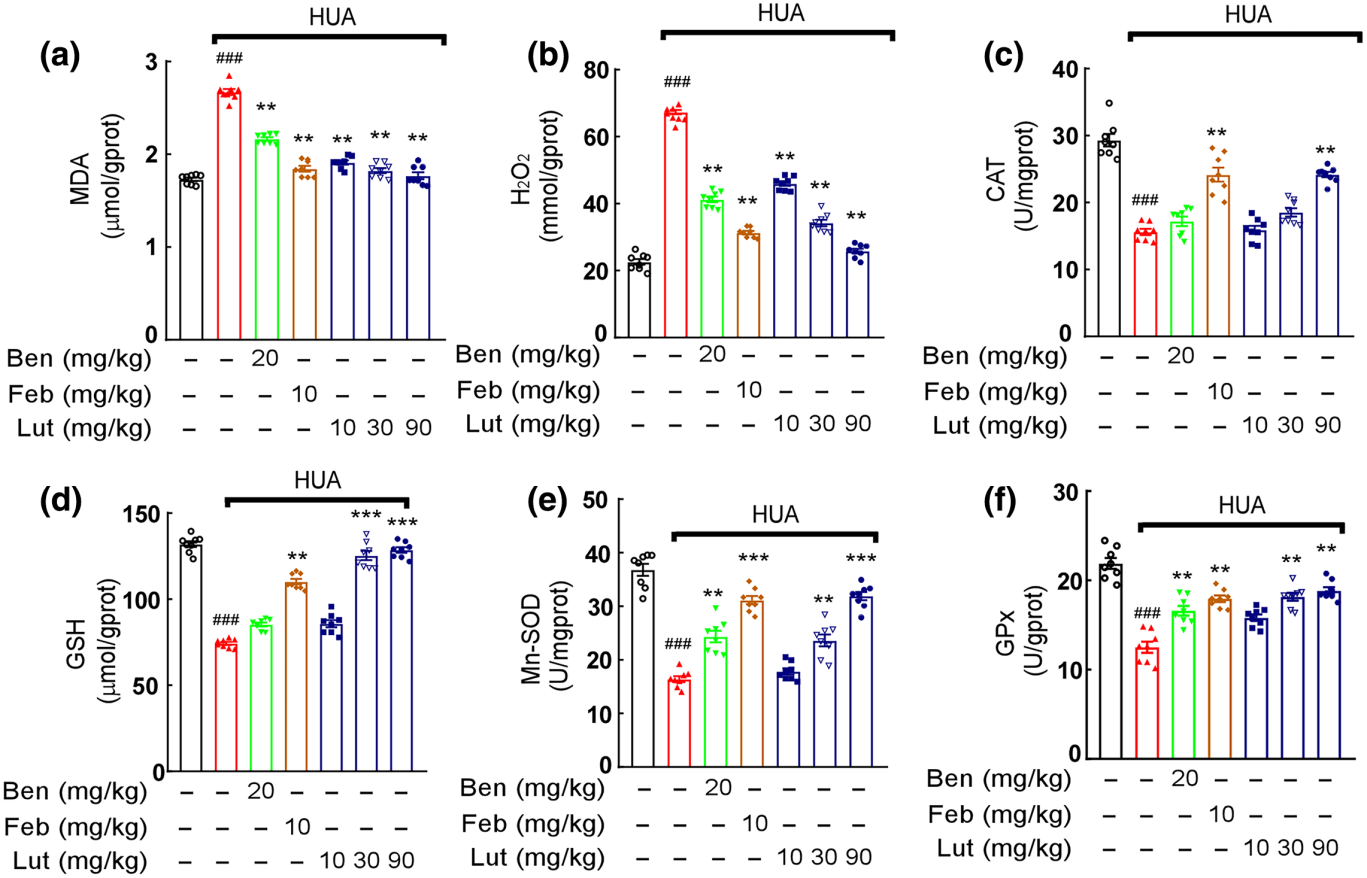
Luteolin alleviates renal oxidative stress in mice with hyperuricemic nephropathy. (a) Kidney tissues were homogenized for evaluating MDA contents. (b) H_2_O_2_ contents. (c) Enzymatic activity of CAT. (d) GSH contents. (e) Enzymatic activity of Mn‐SOD. (f) Enzymatic activity of GPx. Data were means ± *SD* (*n* = 8). ^###^ *p* < .001 vs. control group. **p < .01, ***p < .001 vs. HUA model group.

The production of UA by XO was accompanied by free radical production, which can cause renal oxidative stress. As predicted, renal malondialdehyde (MDA) levels and serum H_2_O_2_ concentrations were abnormally increased in HUA mice and significantly inhibited by the effect of luteolin (Figure 4a,b). The effect of luteolin on XO‐mediated renal oxidative stress was further assessed by measuring CAT, GSH, Mn‐SOD, and GPx levels. The lower levels of CAT, GSH, Mn‐SOD, and GPx in HUA mice compared to controls were significantly improved after 2 weeks of luteolin treatment (Figure 4c–f). These findings demonstrated that luteolin attenuates renal oxidative stress in urate nephropathy.

### 3.5 Luteolin alleviates renal oxidative stress in mice with hyperuricemic nephropathy via activation of the renal SIRT1/6 cascade and its downstream Nrf2‐mediated antioxidant pathway

**FIGURE 5:**
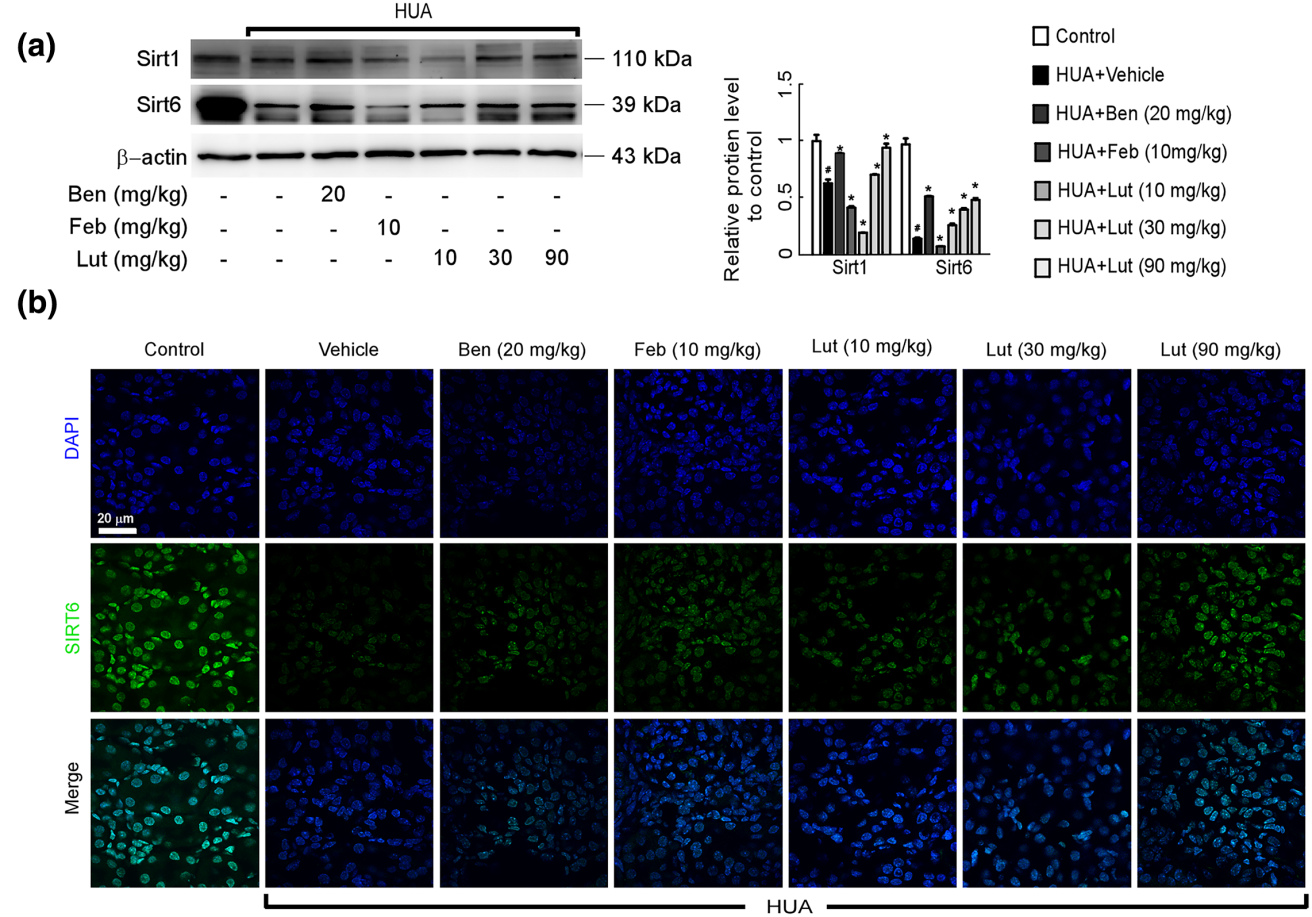
Luteolin up‐regulated protein expression level of Sirt1 and Sirt6 in the kidney of hyperuricemia mice. (a) Protein expression levels of Sirt1 and Sirt6 in kidney were determined by western blots (left) and quantifications (right), which were normalized with *β*‐actin. Data were means ± *SD* (*n* = 3). ^#^ *p* < .05 *vs*. control group. *p < .05 *vs*. HUA model group. (b) Photomicrographs of Sirt6 immunofluorescence stained kidney tissues in control and hyperuricemia mice with or without luteolin treatment (magnification 1000×).

The Sirtuin group plays a crucial role in multiple physiological processes, including cellular metabolism, the aging process, programmed cell death, the body's response to inflammation, and the impact of oxidative stress. The aim of this research was to investigate whether luteolin could alleviate oxidative stress in the kidneys of mice suffering from hyperuricemic nephropathy by activating various members of the sirtuin family specifically found in the renal system. The Western blot analysis revealed that the levels of Sirt1 and Sirt6 protein expression in the kidney of HUA mice were notably reduced compared to the normal controls. However, the presence of luteolin significantly enhanced the expression of both Sirt1 and Sirt6 proteins, leading to a dramatic increase. A dose‐dependent effect was observed (Figure 5a). In addition, we further examined the expression of Sirt6 by immunofluorescence staining. Sirt6 was positioned within the nucleus of renal tubular cells. Micrographs showed markedly higher protein expression level of Sirt6 in the luteolin group compared to the HUA model group (Figure 5b). These results are consistent with those found in Western blot.

**FIGURE 6:**
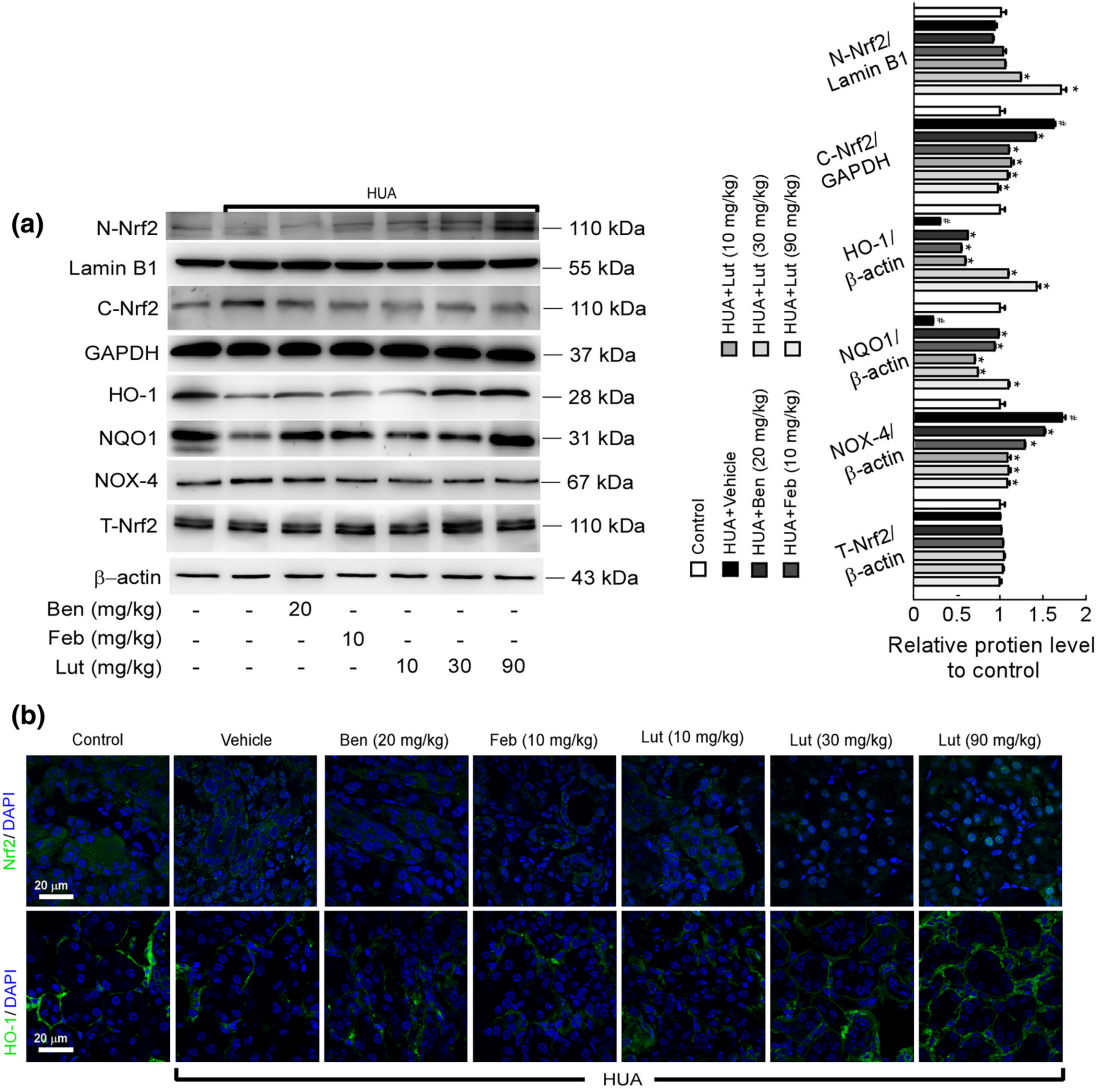
Luteolin inhibited oxidative stress in the kidney of hyperuricemia mice by activating the Nrf2/HO‐1 signaling pathway. (a) Expression levels of Nrf2/HO‐1 signaling pathway‐related protein in the kidney were determined by Western blots (left) and quantifications (right), which were normalized with *β*‐actin. Data were means ± *SD* (*n* = 3). ^#^ *p* < .05 *vs*. control group. *p < .05 *vs*. HUA model group. (b) Photomicrographs of Nrf2 and HO‐1 immunofluorescence stained kidney tissues in control and hyperuricemia mice with or without luteolin treatment (magnification 1000×).

Sirt1 and Sirt6 play a significant role in the antioxidant mechanisms induced by nuclear factor‐erythroid 2‐related factor 2 (Nrf2). Nrf2 facilitates phase II detoxification enzyme and antioxidant enzyme expression through redox sensitivity and the presentation of antioxidant enzyme genes after nuclear translocation. Therefore, we immediately assessed the influence exerted by luteolin upon the protein of Nrf2 and related antioxidant enzyme levels. The Western blot findings demonstrated that luteolin successfully decreased the levels of cytoplasmic Nrf2 (C‐Nrf2) and NADPH oxidase 4 (NOX‐4) in the proximal tubules of HUA mouse kidneys, while simultaneously enhancing the intranuclear expression of N‐Nrf2, HO‐1, and NQO1 (Figure 6a). Under normal physiological circumstances, Nrf2 was found in the cytoplasm of the renal proximal tubule cells, whereas HO‐1 was situated in the basement membrane of the proximal tubule. The micrographs of kidney tissue stained with Nrf2 immunofluorescence showed that luteolin enhanced the expression of N‐Nrf2 and HO‐1 in comparison to the model group (Figure 6b). Altogether, these results suggested that luteolin alleviated renal oxidative stress in mice with hyperuricemic nephropathy associated with activating the renal SIRT1/6 cascade response and its downstream Nrf2‐mediated antioxidant pathway.

### 3.6 Luteolin inhibits XO activity in hyperuricemic mice and shows a direct high‐affinity binding to XO in molecular docking analysis

**FIGURE 7:**
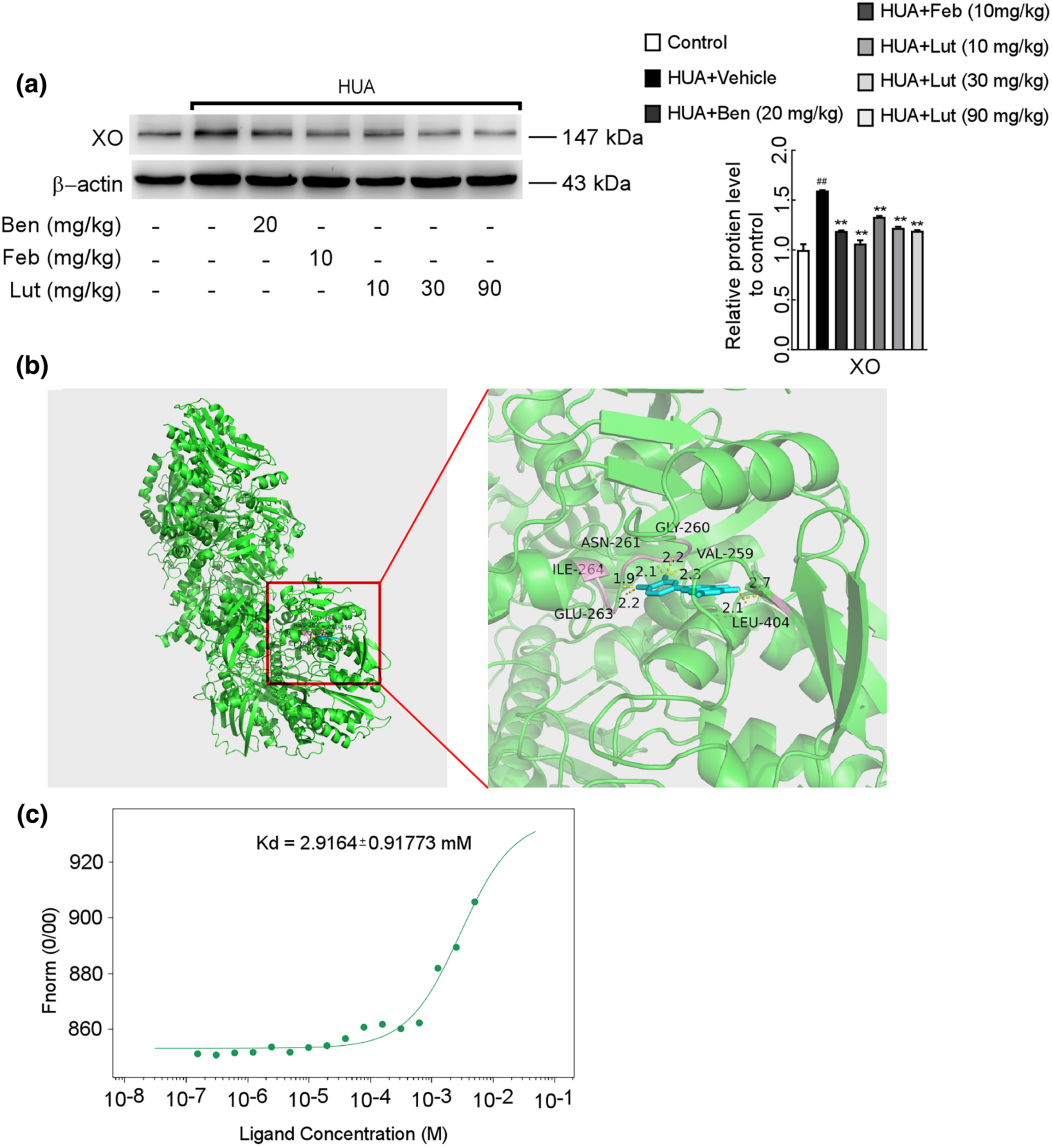
The inhibitory effect of luteolin to XO in hyperuricemia mice in vivo, and the binding affinity of luteolin to XO as determined by molecular docking and MST. (a) Protein expression levels of XO in the liver were determined by Western blots (left) and quantifications (right), which were normalized with *β*‐actin. Data were means ± *SD* (*n* = 3).  ^##^ *p* < .01 *vs*. control group. **p < .01 *vs*. HUA model group. (b) The binding model of cattle (*Bos taurus*) source XO‐luteolin complex established by molecular docking. (c) Steady‐state results of luteolin and XO.

The metabolism of uric acid was reduced in mice with hyperuricemic nephropathy. The metabolic process of uric acid is aided by proteins responsible for uric acid transport and organic anion transporters. In our preceding investigation (Yu et al., 2021), it was noted that luteolin boosts the levels of organic anion transporters associated with the elimination of uric acid in the kidneys of mice with hyperuricemic nephropathy. The XO activity in the liver and serum of HUA mice was higher than in the solvent control. In contrast, the enzymatic activity of XO in the liver and serum of mice was significantly decreased by luteolin at low, medium, and high doses. The article does not present the data. Furthermore, the immunoblotting analysis of proteins also revealed a noteworthy decrease in XO protein expression in the luteolin‐treated group compared to HUA mice, in a manner dependent on the dosage administered (Figure 7a). It indicates that luteolin may reduce uric acid by inhibiting XO activity in HUA mice. Typically, two molecules were considered to have good binding activity when their docking binding energy was less than or equal to −5.0 kcal/mol. The binding energy of luteolin and XO was found to be −9.7 kcal/mol by molecular docking (Figure 7b). The possible binding sites corresponded to six hydrogen bonds such as ASN‐261, GLY‐260, VAL‐259, LEU‐404, GLU‐263, and ILE‐264. To further evaluate the affinity between purified XO and luteolin, we performed microscale thermophoresis tests (MST), which were used to analyze the interactions between biomolecules. In MST, the equilibrium dissociation constant (Kd) was used to show the intermolecular affinity. The Kd value of luteolin is 2.9 mmol/L, which binds directly to XO protein, and the binding rate of luteolin to XO is more than 95% (Kd = A*B/AB) (Figure 7c). It seems that the binding of luteolin to XO is specific and stable.

### 3.7 Luteolin reduces uric acid levels in adenosine‐induced hyperuric acid cell model

**FIGURE 8:**
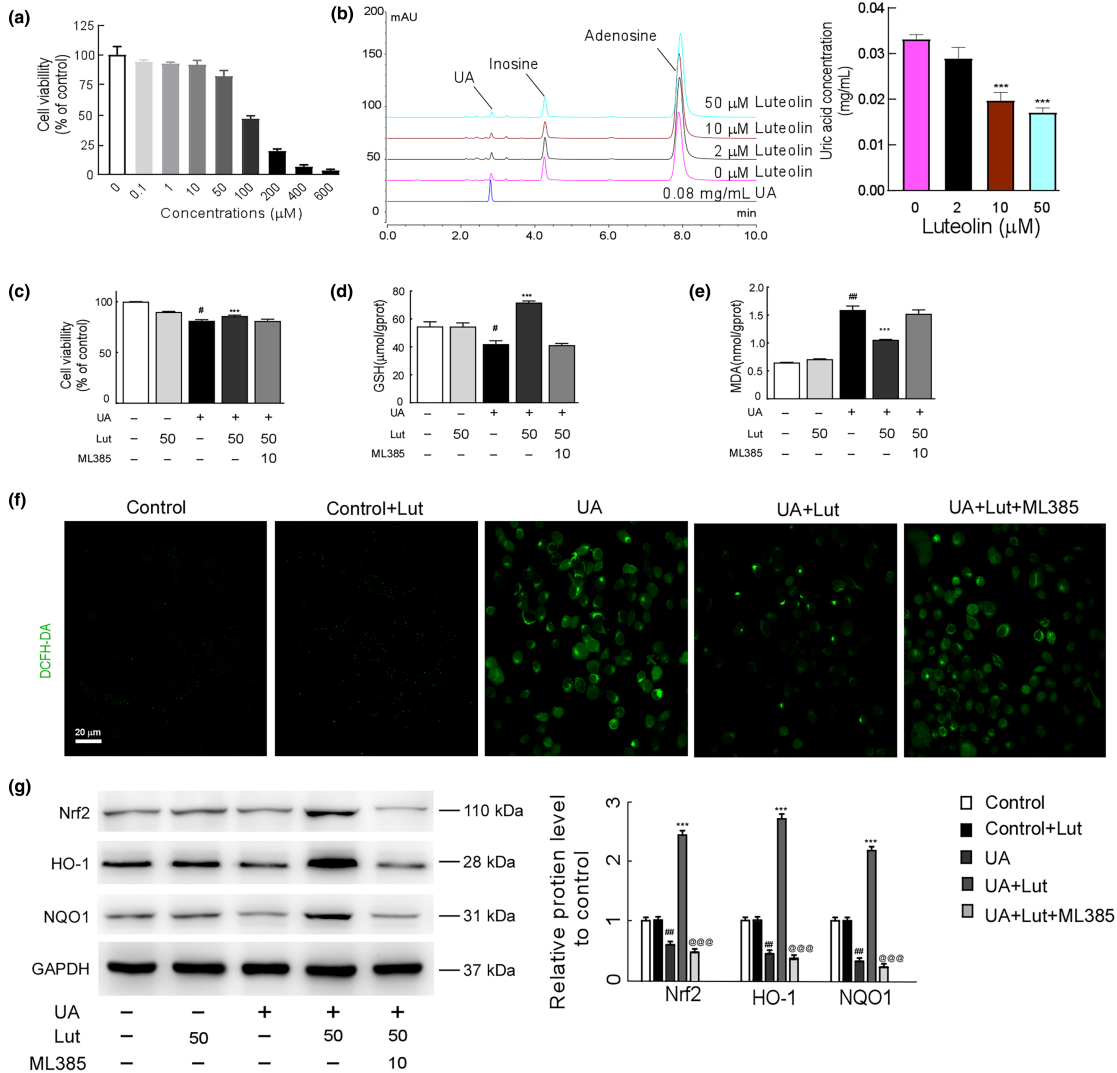
Luteolin reduces uric acid levels in adenosine‐induced hyperuric acid cell model and Nrf2 deficiency attenuated luteolin efficiency in restoring oxidative balance in uric acid–induced hyperuric acid cell model. (a) Effect of luteolin on NRK52E cell viability. (b) HPLC detection of luteolin on the level of uric acid in the cell culture supernatant of hyperuricemia cell model (254 nm), left qualitative results, right quantitative results. Effect of luteolin and or ML385 on cell viability (c) GSH (d), MDA (e), ROS (f), and Nrf2‐related protein (g) level in hyperuricemic cell model. Means ± *SD* (*n* = 6 for a–e; *n* = 3 for f–g). ^#^ *p* < .05, ^##^ *p* < .01, ^###^ *p* < .001 *vs*. control group. ***p < .001 *vs*. UA model group, ^@@@^ *p* < .001 *vs*. UA + Lut group.

To confirm the impact of luteolin on uric acid, the NRK52E cell model with adenosine‐induced hyperuricemia was created for in vitro experiments. The cytotoxicity assay was performed using different luteolin concentrations to determine the appropriate intervention dose of luteolin on the hyperuricemic cell model. Different concentrations (0, 0.1, 1, 10, 50, 100, 200, 400, 600 μmol/L) of luteolin showed elevated cell viability of NRK52E cells (Figure 8a). The IC_50_ value of luteolin on NRK52E cells was 97.85 μM. In the hyperuricemic cell model, treatment with 50 μmol/L luteolin did not considerably affect NRK52E cell viability, so 2, 10, and 50 μmol/L luteolin were chosen to intervene in the hyperuricemic cell model in the following experiments. HPLC was used to measure the uric acid levels in the supernatant of the cell culture for both the luteolin and hyperuricemic cell models. The results suggested that luteolin reduced uric acid levels dose‐dependently (Figure 8b).

### 3.8 Nrf2 deficiency attenuated luteolin efficiency in restoring oxidative balance in uric acid–induced hyperuric acid cell model

ML385 (an inhibitor of Nrf2) was employed to demonstrate that the protective effect of luteolin against HUA‐induced kidney injury operates through an Nrf2‐dependent mechanism. Luteolin improved the decrease in cell viability and GSH induced by high UA, but ML385 attenuated the impact of luteolin (Figure 8c,d). Likewise, the decrease of MDA and ROS levels by luteolin was also blocked after ML385 treatment (Figure 8e,f). We measured the levels of protein expression for Nrf2, as well as its downstream signals NQO1 and HO‐1. Luteolin significantly improved protein expression levels of Nrf2, NQO1, and HO‐1. However, ML385 blunted the effect of luteolin (Figure 8g).

## 4 DISCUSSION

In most animals, uric acid is decomposed into allantoin, a highly soluble compound that can be eliminated through urine. Due to evolutionary genetic mutations, humans are unable to oxidize uric acid, causing the body to fail to break down uric acid into the soluble compound allantoin, ultimately maintaining blood uric acid at a relatively constant level. Steady uric acid levels are an evolutionary choice to satisfy the basic needs of human survival. However, excessive uric acid is detrimental to human health (Alvarez‐Lario & Macarron‐Vicente, 2010; Maiuolo et al., 2016). In addition to affecting quality of life, hyperuricemia can lead to complications such as gouty arthritis, obesity, hypertension, acute or chronic renal failure, heart failure, and acute ischemic stroke (Borghi et al., 2020; Sharaf El Din et al., 2017). Promoting a healthy diet and regular exercise are essential for treating HUA, while also playing a crucial role in the promotion of uric acid metabolism and the inhibition of uric acid production. The primary objective of medications for HUA is to hinder the production of uric acid or enhance its metabolism to sustain the equilibrium of uric acid. In the present study, luteolin, a natural flavonoid with a high medicinal value, was found to promote uric acid excretion and improve hyperuricemia‐induced kidney injury and apoptosis. Luteolin also enhanced the levels of uric acid transporter proteins and triggered the renal SIRT1/6 cascade along with its subsequent Nrf2‐mediated antioxidant pathway. In addition, luteolin inhibited hepatic XO activity. To the best of our knowledge, this study is the first to show that luteolin can combat HN by enhancing uric acid metabolism in the kidneys, activating the antioxidant system, and suppressing xanthine oxidase activity, providing critical molecular evidence that luteolin regulates renal uric acid transporter proteins, activates endogenous antioxidant pathways, and inhibits XO activity.

An efficient and stable animal model of hyperuricemia is essential to develop drugs for HN. A combination of xanthine and PO was used to create a mouse model of hyperuricemia‐induced nephropathy. XO converts xanthine to uric acid, thereby increasing the uric acid source and maintaining consistently high uric acid levels in the bloodstream of mice. Although luteolin components isolated from various plants such as celery seeds, *Gnaphalium affine* D., and *DonLychnophora trichocarpha* Spreng. have demonstrated the capacity to notably decrease levels of blood uric acid in hyperuricemia rodent models. However, there is a lack of comprehensive research on its safeguarding impact on the kidney in hyperuricemic nephropathy (de Souza et al., 2012; Lin et al., 2018; Zhang et al., 2022). In our study, we found that luteolin not only had a notable uric acid‐lowering effect but also significantly improved tubular dilation and epithelial atrophy in the kidneys of mice with HN. Notably, luteolin effectively suppressed Kidney injury molecule 1 (KIM‐1) and the mitochondrial apoptotic pathway. These studies showed that luteolin reduces uric acid levels and considerably improves tubular dilation and epithelial atrophy in the kidneys of mice with HN. Notably, kidney injury molecule 1 (KIM‐1) and the mitochondrial apoptotic pathway were significantly inhibited by lignocaine. These results suggest that luteolin effectively alleviates kidney injury and apoptosis in mice with HN.

Uric acid possesses both antioxidant and pro‐oxidant characteristics (Gherghina et al., 2022; Liu et al., 2021). Normal UA levels demonstrate strong antioxidant properties, protect neurons, relieve neurological disorders, and contribute 55% of the extracellular capacity to counteract free radicals (Alvarez‐Lario & Macarron‐Vicente, 2010; Parkinson Study Group et al., 2021; Waring, 2002; Yu et al., 1998). Increased levels of uric acid in the bloodstream are closely linked to oxidative stress injury, which triggers the activation of nicotinamide adenine dinucleotide phosphate (NADPH) oxidase, an essential enzyme that controls the rate of reactive oxygen species (ROS) generation (Khosla et al., 2005; Liu et al., 2021). Mitochondria are another important source of ROS. Mitochondria serve as focal points for intracellular energy metabolism and play crucial roles in oxidative phosphorylation. They facilitate the transfer of electrons from complexes in the electron transport chain to free radicals, thereby generating ROS (Sinha et al., 2013). In a previous study, long‐term hyperuricemia was found to result in impaired mitochondrial function in the kidneys, which is linked to oxidative stress and damage to the tubules in the cortex of rat kidneys (Cristobal‐Garcia et al., 2015). Additionally, hyperuricemia mediates mitochondrial calcium overload, leading to mitochondrial Na/Ca exchange dysfunction and increased ROS production (Su et al., 2020). Renal mitochondrial dysfunction ultimately triggers pathological changes, such as DNA damage, enzyme oxidation and inactivation, inflammatory cytokine production, and apoptosis. Herein, a significant increase in HUA‐induced ROS content and the activation of mitochondrial apoptosis were demonstrated in in vitro and in vivo. Furthermore, as a consequence of HUA‐induced renal dysfunction, increases in renal malondialdehyde (MDA), serum H_2_O_2_, and other critical oxidative stress indicators were observed in HUA mice, which were effectively reversed after luteolin treatment. This suggests that treatment with luteolin for 2 weeks can alleviate oxidative stress and improve renal function in HN mice.

Exploring the mechanism by which luteolin inhibits oxidative stress in the kidneys of mice with HN is essential for understanding its resistance to HN. The Keap1‐Nrf2 pathway, a classical antioxidant signaling pathway, plays a crucial role in combating ROS generated by mitochondria. During oxidative stress, Nrf2 is separated from Keap1 (Hayes & Dinkova‐Kostova, 2014). It attaches to the antioxidant response element (ARE) promoter, a process during which Sirt1 and Sirt6 are highly involved, ultimately promoting the expression of critical genes encoding antioxidants and phase II detoxification enzymes (Nguyen et al., 2009; Pan et al., 2016; Yang et al., 2016). The flavonoid luteolin is a natural antioxidant. Numerous in vitro and in vivo studies have documented the robust pharmacological properties of luteolin in stimulating Nrf2 activation (Chen et al., 2022; Li et al., 2019; Z. H. Zhang et al., 2021). Furthermore, luteolin exerts nephroprotective effects by alleviating oxidative stress, reducing inflammatory responses, and inhibiting apoptosis (Liu et al., 2017; Owumi et al., 2021). By investigating the impact of luteolin on Nrf2 and oxidative stress, we investigated the antioxidant mechanism of luteolin in HN kidney tissues. Luteolin promoted the translocation of Nrf2 to the nucleus by increasing the expression of SIRT1 and STRT6 and the GSH, NQO1, and HO‐1 levels, as well as CAT, SOD, and GPx activity, indicating that the renoprotective effects of luteolin in HUA mice may be achieved by activating the Nrf2‐mediated antioxidant response.

An essential trigger for HN is high uric acid levels in the blood. Excess uric acid forms monosodium urate crystals, which are deposited in the urinary system of the kidney, causing substantial damage. Similar to previous studies, our findings provide direct evidence for the uric acid‐lowering effect of luteolin. Based on this, we explored the molecular mechanisms underlying its uric acid‐lowering effect. Our previous results suggest that luteolin increases ABCG2, OAT1, and OAT3 expression to promote UA excretion. Since XO is the rate‐limiting enzyme in purine metabolism and is responsible for SUA balance (Gliozzi et al., 2016), we investigated whether luteolin acts as a potent inhibitor of XO. In our previous study, XO activity was reduced in both the liver and serum of luteolin‐treated HUA mice. In vitro experiments demonstrated that luteolin binds directly to XO, providing molecular evidence for the luteolin‐induced reduction in uric acid levels.

## 5 CONCLUSIONS

In summary, our current work emphasizes that luteolin can attenuate renal injury in hyperuricemic nephropathic mice by modulating uric acid transporters, inhibiting XO activity, and activating the renal SIRT1/6 cascade and its downstream Nrf2‐mediated antioxidant pathway. Noticeably, luteolin exerted nephroprotective effects without significant hepatotoxicity compared to conventional therapeutic agents. In our previous investigation (Yu et al., 2021), it was observed that the administration of luteolin did not yield any substantial effects on liver function indexes and liver histopathology in hyperuricemic mice. The findings indicate that luteolin, a naturally occurring functional substance, could potentially serve as an innovative therapeutic approach for addressing hyperuricemia (HUA) and kidney insufficiency. Further research is required to assess the mechanism of action and targets of luteolin in both uric acid‐induced acute kidney injury and chronic kidney disease.

## AUTHOR CONTRIBUTIONS

**Huifan Yu:** Conceptualization (equal); data curation (equal); formal analysis (equal); funding acquisition (equal); investigation (equal); methodology (equal); writing – original draft (equal). **Linsheng Huang:** Investigation (equal). **Lili Gui:** Investigation (equal). **Zhengkun Wu:** Investigation (equal). **Han Luo:** Investigation (equal). **Mao Xu:** Investigation (equal). **Yan Zhang:** Investigation (equal). **Yongshuai Qian:** Investigation (equal). **Wenjie Cao:** Investigation (equal). **Li Liu:** Investigation (equal). **Fei Li:** Conceptualization (lead); funding acquisition (lead); investigation (lead); supervision (lead); writing – review and editing (lead).

## FUNDING INFORMATION

This project was supported by research funds from the Natural Science Foundation of Hubei Provincial Department of Education (Grant No. D20192101, Q20222102), the Cultivating Project for Young Scholar at Hubei University of Medicine (2021QDJZR009), the Biomedical Research Foundation, Hubei University of Medicine (Grant No. HBMUPI201805), the Advantages Discipline Group (Biology and Medicine) Project in Higher Education of Hubei Province (2021–2025) (Grant No. 2023BMXKQT1), and the Postgraduate Science and Technology Innovation Project (Grant No. YC2023067, YC2022023, YC2020040).

## CONFLICT OF INTEREST STATEMENT

All authors have no conflicts of interest.

## ETHICS STATEMENT

The animal study was reviewed and approved by the Institutional Animal Care and Use Committee of Hubei University of Medicine.

## Supporting information


Table S1.

## Data Availability

Data are available by contacting the corresponding author (Prof. Fei Li).
