# Supplementary material for: Luteolin ameliorates hyperuricemic nephropathy by activating urate excretion and Nrf2/HO‐1/NQO1 antioxidant pathways in mice
Source: Food Sci Nutr. 2024 Aug 20;12(10):8053–66. doi: 10.1002/fsn3.4403 (PMC11521689; doi:10.1002/fsn3.4403)
Supplement: Supplementary file 1 — Table S1. [file FSN3-12-8053-s001.docx]

**Supplementary Table 1 All antibodies used in this study.**

| **Antibody Name** | **Manufacturer** | **Product number** |
| --- | --- | --- |
| KIM-1 | abcam | ab78494 |
| Bax | abcam | ab32503 |
| Bad | abcam | ab32445 |
| Bcl-xL | abcam | ab32370 |
| Bcl-2 | CST | #4223 |
| caspase-3 | abcam | ab184787 |
| caspase-7 | abcam | ab181579 |
| caspase-10 | abcam | ab2012 |
| Nrf2 | abcam | ab137550 |
| Lamin B1 | abcam | ab65986 |
| HO-1 | abcam | ab13248 |
| NQO1 | abcam | ab34173 |
| SIRT1 | abcam | ab110304 |
| SIRT6 | abcam | ab191385 |
| OCT1 | Biosynthesis | bs-20814R |
| OCT2 | abcam | ab170871 |
| NOX-4 | abcam | ab154244 |
| XO | abcam | ab231316 |
| β-actin | CST | #4970 |
| GAPDH | abcam | ab8245 |
| Goat Anti-Rabbit IgG H&L (HRP) | abcam | ab97051 |
| Goat Anti-Mouse IgG H&L (HRP) | abcam | ab97023 |
| Donkey Anti-Mouse IgG H&L (Alexa Fluor 594) | abcam | ab150108 |
| Goat Anti-Rabbit IgG H&L (Alexa Fluor 488) | abcam | ab150077 |
| Goat Anti-Rabbit IgG H&L (Alexa Fluor 594) | CST | 8889S |
| Goat Anti-Mouse IgG H&L (Alexa Fluor 488) | abcam | ab150113 |
